# Personality of Public Health Organizations’ Instagram Accounts and According Differences in Photos at Content and Pixel Levels

**DOI:** 10.3390/ijerph18083903

**Published:** 2021-04-08

**Authors:** Yunhwan Kim, Sunmi Lee

**Affiliations:** 1 College of General Education, Kookmin University, Seoul 02707, Korea; yunhwankim2@kookmin.ac.kr; 2 Department of Applied Mathematics, Kyung Hee University, Yongin 17104, Korea

**Keywords:** Instagram, public health, organization, personality traits, Microsoft Azure Cognitive Services, IBM Watson Personality Insights, pixel

## Abstract

Organizations maintain social media accounts and upload posts to show their activities and communicate with the public, as individual users do. Thus, organizations’ social media accounts can be examined from the same perspective of that of individual users’ accounts, with personality being one of the perspectives. In line with previous studies that analyzed the personality of non-human objects such as products, stores, brands, and websites, this study analyzed the personality of Instagram accounts of public health organizations. It also extracted features at content and pixel levels from the photos uploaded on the organizations’ accounts and examined how they were related to the personality traits of the accounts. The results suggested that the personality of public health organizations can be summarized as being high in openness and agreeableness but lower in extraversion and neuroticism. Openness and agreeableness were the personality traits associated the most with the content-level features, while extraversion and neuroticism were the ones associated the most with the pixel-level features. In addition, for each of the two traits associated the most with either the content- or pixel- level features, their associations tended to be in opposite directions with one another. The personality traits, except for neuroticism, were predicted from the photo features with an acceptable level of accuracy.

## 1. Introduction

Personality has received a lot of research attention concerning individuals’ use of social media. Since personality is a theoretical concept which covers the behavioral phenotypes of individuals and their variation within populations [1], it has been applied to understand how individuals differ in terms of their social media use behavior [2,3]. In addition, another focus has been placed on how the characteristics of the posts uploaded by individuals differ with regard to their personality [4,5]. This investigation was mainly carried out in one of the two ways following: revealing the difference in posts by uploaders’ personality [6,7] or predicting uploaders’ personality using features of posts [8,9].

The social media accounts of organizations, however, have not received much research attention in terms of the relationship between their personality and the characteristics of the posts uploaded on the accounts. One may think of personality as being peculiar to human beings, but a body of literature has investigated the personality of non-human objects, such as products, stores, brands, and websites, to understand their appearances and behaviors [1,10,11]. This attempt can be extended to examining the personality of organizational social media accounts. In addition, organizational accounts are basically the same as individual users’ accounts in social media space—they can upload posts on their accounts, become friends with or follow other accounts, and like and make comments to other users’ posts. Thus, organizations can express themselves in the same way that individuals do in social media, and the personality of organizational accounts can be examined in the same way as that in examining individual users’ accounts [12].

Meanwhile, photos in social media have not been actively investigated in terms of their relationships with the personality of uploaders. The posts in text form still take the largest share in social media, and many previous studies have analyzed text data to examine the relationships between their characteristics and the personality of uploaders [13,14]. Yet, the share of visual data including photos has been rapidly increasing in social media. Photo-uploading functions are provided in existing social media sites such as Facebook and Twitter and photo-centric social media services such as Instagram have become popular, while many studies have been conducted about Instagram in various fields [15,16,17,18]. In this vein, the relationships between social media photo features and uploaders’ personality have been examined in previous research [5,19], but only limited kinds of photos such as profiles or selfies were analyzed in those studies. It needs to be noted also that photos have low (pixel)-level features, as well as high (content)-level features, through which information can be conveyed and meaning can be created. Although pixel-level features were used to analyze the social media photos on individual users’ accounts in previous studies [8,20,21], they were hardly ever used to analyze the social media photos on organizational accounts in terms of their relationships with the accounts’ personality.

Based on these considerations, the objective of this study was to present the personality of Instagram accounts run by public health organizations. Among the huge variety of organizations, this study paid attention to public health organizations. This is because they usually maintain their social media communication focused on their purpose of establishment with little consideration of external conditions. It is less the case with other kinds of organizations, such as political parties or businesses, where keen attention should be kept on the surrounding conditions including the political or market situations and the strategy of competing organizations. In addition, the present research utilized an online artificial intelligence (AI) service to measure the personality of organizational accounts using the text part of posts uploaded on the accounts. This method of measuring personality using online data is reported to be the same or more accurate than using self-reported data [22,23] and has been used in previous research [12,24,25,26,27,28]. Another objective of this study was to investigate how the uploaded photos are different in terms of their high- and low-level features by the accounts’ personality. Literature has shown that individual users’ social media content is different with regard to their personality (refer to Section 2.1). This study aimed to examine whether this is also the case concerning the organizational accounts.

The following research questions were raised:

RQ1. What are the characteristics of the personality of public health organizations’ Instagram accounts?

RQ2. What are the relationships between the personality of public health organizations’ Instagram accounts and the features of the photos uploaded on the accounts?

The remainder of this paper is organized as follows. First, previous studies about personality and social media content, inferring personality of non-human objects, and social media content of public health organizations’ accounts are reviewed. Next, comes a description of how the data was gathered, how personality of organizational accounts were measured, and which photo features were used for analysis. Finally, the results of analysis are presented and their implications discussed.

## 2. Related Works

### 2.1. Personality of Social Media Users and According Differences in Content

The Big-Five personality model [29,30] is one of the most widely used frameworks to investigate the difference in social media content. Pentina and Zhang [31] examined whether social media users were different in emotional disclosure with regard to their personality. Their results suggested that extraversion, agreeableness, and conscientiousness were significantly related to revealing positive emotions on Facebook. Miller [32] showed that conscientiousness was negatively related to posting inappropriate materials on Facebook among college students. Wang and Chen [33] analyzed the social media posts uploaded by CEOs to investigate the impact of their personality on business performance. Their results indicated that extraversion, emotional stability, and agreeableness were positively associated with cost efficiency and profitability, while conscientiousness were negatively associated with them. Agarwal and Toshniwal [34] analyzed tweets under various natural hazards conditions and found that Twitter users with a higher level of extraversion and agreeableness uploaded more tweets of leadership concerning behaviors under crisis situations.

The significant association between personality and social media content was also obtained in literature that analyzed photo data. Liu et al. [35] examined the difference in Twitter profile photos by the personality of account owners: their results showed that more positive emotions were revealed in the profile photos of the users with higher levels of agreeableness and conscientiousness, and more aesthetic photos were used as a profile by the users with a higher level of openness. Sorokowska et al. [7] showed that the level of extraversion was significantly associated with the frequency of selfie-posting on various social media. Samani et al. [36] analyzed the Twitter and Flick photos and found that conscientiousness and openness were more predictable than other personality traits from the photos that users uploaded on social media.

In this study, we apply this approach for analyzing organizational social media accounts. While individual users’ accounts have been analyzed in terms of the owners’ personality and their content, it has rarely been investigated whether the content of the posts uploaded on organizational social media accounts is related to the personality of the accounts. Also, this study analyzes all photos uploaded on a given account unlike most of the literature about personality and social media content where only particular kinds of photos such as profiles or selfies have been analyzed—the rare exceptions that have analyzed all photos on a given account include Ferwerda et al. [8] and Kim and Kim [16].

### 2.2. Inferring Personality of Non-Human Objects

Personality can be defined as stable differences in behavioral patterns among individuals [37]. In other words, while a variety of traits can be used for its judgement, personality is categorization of behaviors manifested outwardly based on the reasoning by oneself or external observers [1]. Based on this definition of personality which centers on the reasoning based on behaviors, we can infer the personality of the non-human objects which show stable behavioral patterns.

First, the personality of products was investigated based on the assumption that each product has a personality which differentiates itself from other products [10]. This product personality was reported to have an effect on consumers’ preference of the product [38,39]. Next, the personality of stores was examined. For example, d’Astous and Lévesque [40] devised a scale for measuring store personality and showed that it consisted of five dimensions, namely sophistication, solidity, genuineness, enthusiasm, and unpleasantness. Also, the personality of brands was investigated. While there is still a debate as to whether a scale for human personality can be used for measuring different types and contexts of brands [41], previous studies either employed the personality traits of humans, like the Big-Five model, to understand brand personality [42,43] or showed the correspondence between brand and human personalities [44,45]. These attempts to investigate the personality of non-human objects were also made concerning the online objects without real-world existence, such as e-brand [11].

Furthermore, the personality of websites has been investigated. Chen and Rogers [46] and Poddar et al. [47] suggested the concept of website personality, that is an extension of store and brand personalities, and developed the Website Personality Scale for its measurement. Shobeiri et al. [48,49] used this concept and measurement scale to investigate the personality of e-retailer’s websites. They showed that, among the five dimensions of website personality, enthusiasm had a positive influence, while unpleasantness had a negative influence on involvement and attitude toward the sites. Akrimi and Khemakhem [50] and Akrimi [51] examined the website personality of internet service providers to show that enthusiasm and genuineness dimensions were positively associated with satisfaction while solidity and unpleasantness dimensions were negatively associated with it. Rezaei et al. [52] investigated the personality of tourism products websites and revealed that the personality had an influence on utilitarian browsing, hedonic browsing, and impulse buying. Jain and Yadav [53] showed the positive impact of website personality on individual users’ purchase intention in online stores.

While personality has been investigated in terms of a variety of non-human objects, as seen from the above, the personality of organizational accounts in social media has been relatively understudied. According to the CASA (Computers Are Social Actors) perspective, users perceive computers as being like humans and apply human social norms when they interact with computers, thus computer personalities are accepted as psychologically real to users [54]. In this vein, the personality of organizational accounts in social media can be accepted as real and similar to that of individual users’ accounts. Thus, we can infer the personality of organizational accounts in the same way that we infer personalities of humans and other objects. Here, online AI services can be useful; the personality of an author can be estimated based on the text that he or she wrote. Previous studies reported that personality can be judged with equal or even more accuracy by analyzing web-logs using computers than by human observers [22,23,55,56], and many studies have judged the personality of authors of various texts using online AI services [12,24,25,27,28]. In the line of this research, the present study measured the personality of organizational Instagram accounts using an online AI service and investigated how their personality is related to the features of their photos at content and pixel levels.

### 2.3. Content of Public Health Organizations’ Social Media Accounts

Organizations may maintain social media accounts having particular purposes in mind. Public health organizations, for example, may have their own strategic goals and may consider communicating with the public via social media as one of the ways that they achieve these goals [57]. Thus, they may publish posts which are closely related to their goals, and we can understand how they communicate with the public by analyzing the content of the posts uploaded on the organizational accounts.

Based on this consideration, previous studies analyzed the content of public health organizations’ social media accounts. Guidry et al. [58] investigated how health organizations address the health crisis in their social media communication. The results of analyzing Ebola-related social media posts suggested that social media messaging can be most effective when the messages were solution-based, contained visual imagery, and acknowledged public fears and concerns. Steffens et al. [59] examined the health organizations’ response to misinformation concerning vaccination on social media. They identified organizations’ strategies to face the challenges of misinformation; communicating with openness, encouraging audience dialogue, building community partnerships, and countering misinformation with care. Chen et al. [60] analyzed the social media messages from Chinese national health organization during the COVID-19 crisis and found that posts about the latest news and government’s effective handling of the crisis drew more engagement from the public. Liao et al. [61] also investigated social media communication during the COVID-19 crisis; their results showed that most of the posts from government health organizations were about epidemic situations, information about the new disease, and official actions.

While many studies, including the ones briefly reviewed above, have focused on the content of social media posts uploaded by public health organizations, most of them analyzed the text data. In comparison, the photos uploaded on public health organizations’ social media accounts have not drawn much attention in the literature. The work of Kim and Kim [62] is one of the rare exceptions—they analyzed the Instagram photos on the US Centers for Disease Control and Prevention (CDC) account. Yet, although their work investigated how photos were used for public health communication, it is of limited value in that their analysis was confined to the data from a single organization. The present study tries to overcome this limitation by analyzing the Instagram photos uploaded on 265 public health organizations’ accounts.

## 3. Method

### 3.1. Research Sample

To obtain the list of public health organizations, we visited the webpage of organizations list in US Department of Health and Human Services (https://healthfinder.gov/FindServices/Organizations/default.aspx; accessed on 1 November 2019). The organizations in Federal Agencies, State Health & Human Services, and Nonprofit Organizations categories were included in the research sample. We also visited the official website of each organization to obtain its Instagram account. The organizations which did not have Instagram accounts, or which uploaded less than 30 posts were excluded from the research sample. Also, additional public health organizations which were not in the above list were found and included. As a result, 265 Instagram accounts of public health organizations in total were selected as the research sample (see Table 1). Photos and accompanying data from the accounts were downloaded using Instagram scraper (https://github.com/arc298/instagram-scraper; accessed in 11 November 2019).

### 3.2. Measuring Personality of Organizational Accounts

The personality of organizational accounts was measured using IBM Watson Personality Insights (https://www.ibm.com/watson/services/personality-insights/; accessed in 12 December 2019). The pretrained AI service infers individuals’ personality characteristics from digital communication in text form including email, text messages, tweets, and forum posts by linguistic analytics (https://console.bluemix.net/docs/services/personality-insights/index.html#about; accessed in 12 December 2019). In this study, the text parts of all Instagram posts uploaded on a given account were sent to the server via application programming interface (API), which returned the Big-Five personality traits [25,26]—openness, conscientiousness, extraversion, agreeableness, and neuroticism—of the account by a value between 0 and 1 for each trait.

### 3.3. Instagram Photo Features

Since the aim of this study was to examine whether photo features are related to the organizational accounts’ personality as they were to individual users’ personality, we employed the photo features that had been used to analyze individual users’ Instagram photos in previous studies [8,62,63]. This enabled us to compare the results of this study about organizational accounts with the ones of previous studies about individuals’ accounts. The features were extracted at content and pixel levels—the content-level features were content category and facial features, and the pixel-level features were pixel color features and visual features.

#### 3.3.1. Content Category

For each photo, it was determined as to which category its content belonged using Computer Vision API in Microsoft Azure Cognitive Services (https://azure.microsoft.com/services/cognitive-services/computer-vision/; accessed in 16 February 2020) [64]. A given photo was sent to the server via API and its content was categorized by the pretrained AI service into one of the 15 predetermined classes (*abstract*, *animal*, *building*, *dark*, *drink*, *food*, *indoor*, *others*, *outdoor*, *people*, *plant*, *object*, *sky*, *text*, or *transportation*), and the share of each class, out of all photos on a given account was calculated. Also, the *Gini* coefficient, which shows the degree of concentration (https://en.wikipedia.org/wiki/Gini_coefficient; accessed in 16 February 2020), was measured for the non-diversity in terms of content category of the photos on a given account.

#### 3.3.2. Facial Features

Human faces on a given photo were detected and features regarding the faces were extracted using Face API in Microsoft Azure Cognitive Services (https://azure.microsoft.com/services/cognitive-services/face/; accessed in 16 February 2020). Specifically, (1) the *number of faces* on a given photo was counted, (2) *closeup* was measured by the ratio of the size of the biggest face to the total size of the photo, and (3) *face ratio* was measured by the ratio of the sum of sizes of all faces to the total size of the photo. (4) *age* was measured by average age, and (5) *gender* was measured by the ratio of the number of female faces to all detected faces in a given photo. In addition, the emotions revealed on each face were determined by the pretrained AI service by eight categories so that the sum of all categories on a face became 1. The averages for each of eight emotions on all faces on a given photo were measured; the eight emotions are (6) *anger*, (7) *contempt*, (8) *disgust*, (9) *fear*, (10) *happiness*, (11) *neutral*, (12) *sadness*, and (13) *surprise*. 

#### 3.3.3. Pixel Color Features

Pixels in digital photos contain information which represents visual characteristics like colors; they can be RGB (red, green, blue), HSV (hue, saturation, value), or others depending on the color space model to be used. This pixel-level information was used to extract the following features of a given photo using Python programming language and OpenCV library. 

First, the means and variances across all pixels in a given photo were measured respectively for red, green, and blue [(1) *red mean*, (2) *red variance*, (3) *green mean*, (4) *green variance*, (5) *blue mean*, (6) *blue variance*], and it was also done for saturation and value (i.e., brightness) for each [(7) *saturation mean*, (8) *saturation variance*, (9) *value mean*, (10) *value variance*]. Concerning hue, which is a nominal feature unlike saturation and value, its total range (0 to 179 in OpenCV) was divided into intervals (7, 23, 35, 90, 136, 169) each of which corresponds to each key color: red, orange, yellow, green, blue, and violet [16]. In a given photo, the share of pixels whose hue falls into each color interval was measured [(11) *red share*, (12) *orange share*, (13) *yellow share*, (14) *green share*, (15) *blue share*, (16) *violet share*], and (17) *the share of warm colors* (red, orange, and yellow) and (18) *the share of cold colors* (green, blue, and violet) were also measured. In addition, the number of peaks in the hue histogram [65,66] [(19) *hue peaks*] was measured—a histogram generated from hues in a given photo was smoothed by Kernel Density Estimation, and the number of local maximums of the smoothed histogram was counted [20].

#### 3.3.4. Visual Features

The features that represent the attractiveness of a given photo [67] were extracted. First, it was measured how bright a given photo is [(1) *brightness*] by the average of luminance (Y values in the YUV color space) in the pixels of the photo and it was measured how colorful a given photo is [(2) *colorfulness*] using the means and standard deviations of metrics composed of relative amounts of red, green, and blue values in the pixels [68]. Next, it was measured how much a given photo corresponds to the human perception of reality [69] [(3) *naturalness*] using the proportion of pixels whose saturation and luminance fall in a certain range [67]. (4) *Contrast* represents the relation of local luminance variations to the surrounding luminance, and it was measured as the standard deviation of luminance in pixels divided by the number of pixels [67], and (5) *RGB contrast*, the extension of contrast into the three-dimensional RGB color space, was also measured. (6) *Sharpness* represents a photo’s clarity and level of detail, and it was measured as a function of Laplacian of each pixel’s luminance, normalized by the local average luminance in the surroundings of each pixel [70].

In addition, two features about color were measured. (7) *Color diversity* represents how diverse the colors used in a given photo are, and it was measured by fractal dimension, which has been used as a metric of color diversity in previous studies [20,71] with the box-counting method [72]. (8) *Color harmony* represents how harmonious the dominant colors in a given photo are, and it was measured by the geometric formulations that the dominant colors generate on the color wheel [73]. A hue histogram was generated and smoothed by Kernel Density Estimation, and the highest and the second highest peaks were identified as the top two dominant colors. The internal angle that the two colors make on the color wheel is color harmony [74]. Finally, affections from the PAD model were used to calculate (9) *pleasure*, (10) *arousal*, and (11) *dominance* based on the formula (1) from previous research [75].

Pleasure = 0.69 × Value + 0.22 × Saturation Arousal = −0.31 × Value + 0.60 × Saturation Dominance = −0.76 × Value + 0.32 × Saturation(1)

## 4. Results

### 4.1. Mean Personality Traits of Public Health Organizations

**Figure 1:**
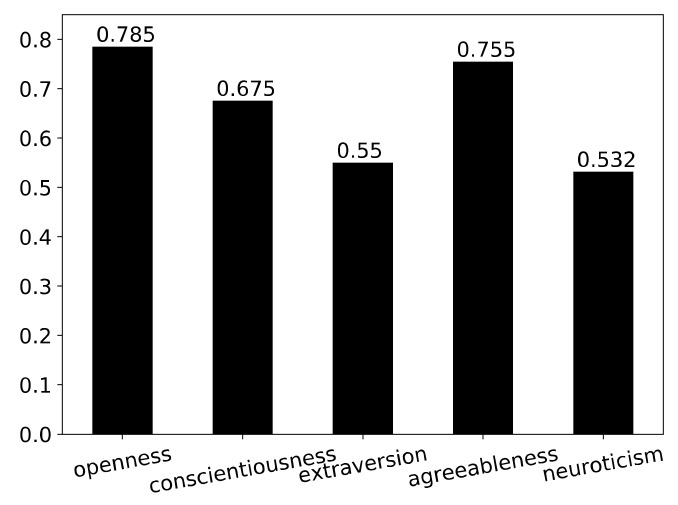
Mean personality traits of public health organizations in the research sample.

The mean personality traits of public health organizations in the research sample are presented in Figure 1 which shows that openness and agreeableness were relatively high in comparison to extraversion and neuroticism. In other words, the personality of public health organizations can be summarized as being high in openness and agreeableness but low in extraversion and neuroticism.

### 4.2. Correlations between Personality Traits and Photo Features

First, the correlations between public health organizations’ personality traits and content category of their Instagram photos were investigated. As shown in Table 2, openness and agreeableness were the personality traits that associated the most with the content category features. Also, a tendency was observed that openness and agreeableness were associated with the content category in opposing directions. For example, the higher share of people photo an account had, the lower the level of openness and the higher level of agreeableness its personality had. The accounts that had a higher share of food photo also showed a higher level of openness and a lower level of agreeableness in their personality. The accounts whose photos were more diverse in content (less Gini) showed a higher level of openness and a lower level of agreeableness.

Next, the correlations between public health organizations’ personality traits and facial features of their Instagram photos were investigated. Table 3 shows, the same as in the content category, that openness and agreeableness were the personality traits that were associated the most with facial features, and their associations were in opposite directions to each other. For example, the more faces an account had on its photos, the lower the level of openness and the higher the level of agreeableness its personality had. The accounts that had older faces on their photos showed a lower level of openness and a higher level of agreeableness in their personality while the happier the faces accounts had on their photos, demonstrated that their personality showed a lower level of openness and a higher level of agreeableness. 

It was investigated how public health organizations’ personality traits were correlated with the pixel color features of their Instagram photos. Table 4 shows that extraversion and neuroticism were the personality traits that associated the most with pixel color features. Also, extraversion and neuroticism tended to show correlations with the pixel color features in opposing directions to each other, although this pattern was less distinct than the ones shown in the content-level features. For example, the accounts with a higher level of extraversion and a lower level of neuroticism uploaded photos with less green mean and blue mean in their pixels while the accounts with a higher level of extraversion uploaded photos with less value mean, but the accounts with a higher level of neuroticism uploaded photos with larger value mean.

Finally, it was examined how public health organizations’ personality traits were correlated with the visual features of their Instagram photos. Table 5 shows, the same as in the pixel color features, that extraversion and neuroticism were the personality traits that associated the most with the visual features. Also, extraversion and neuroticism tended to show correlations with visual features in opposing directions to each other, although this pattern was less distinct than with the ones shown in the content-level features. For example, the photos uploaded on the accounts with a higher level of extraversion were less bright and more natural, while the photos uploaded on the accounts with a higher level of neuroticism were brighter and less natural. In addition, the photos uploaded on the accounts with a higher level of extraversion showed less pleasure and more dominance, while the photos uploaded on the accounts with a higher level of neuroticism showed more pleasure and less dominance.

### 4.3. Predicting Personality Traits Using Photo Features

In addition to the correlational analysis, predictive models were built and analyzed to investigate how accurately the Instagram photo features predicted the personality traits of the organizational accounts. A random forest regressor with 10-fold cross-validation was trained for each personality trait, and a root mean square error (RMSE) was calculated to observe the predictability of each model (see Table 6). Then, we compared these RMSEs with the ones in prior works where social media user personalities were predicted from their photo features—the RMSEs were 0.88 [76], 0.7–0.9 [77], 0.66–0.78 [8], and 0.561–0.737 [16]. Since those studies measured the personality traits using the 5-point Likert scale, we converted their RMSEs into [0, 1] scale used in this study by dividing the value by 4, the range of the 5-point scale. Then, the RMSEs of 0.5–0.7 in the 5-point scale become 0.125–0.175 in the [0, 1] scale.

In comparison with the RMSEs in previous studies, the results in Table 6 suggest that the predictive power of Instagram photo features over the Big-Five personality traits, except neuroticism, of the organizational accounts was acceptable. Neuroticism was reported to be the most difficult personality trait to predict in previous research [78], which is consistent with the result in this study.

## 5. Discussion

Organizations maintain social media accounts and upload posts to show their activities and communicate with the public, as individual users do. Thus, organizations’ social media accounts can be examined from the same perspective as individual users’ accounts, with personality being one of the perspectives. In line with previous studies that analyzed the personality of non-human objects such as products, stores, brands, and websites, this study analyzed the personality of Instagram accounts of public health organizations. It also extracted features at content and pixel levels from the photos uploaded on the organizational accounts and examined how they were related to the personality traits of the accounts. The major findings and discussions about them are as follows.

First, the personality of public health organizations in the research sample can be summarized as being high in openness and agreeableness but low in extraversion and neuroticism. This could be characteristic of public health organizations in comparison with other organizations and individuals whose personalities were measured in previous studies. The personalities of major brands’ Twitter accounts were measured using IBM Watson Personality Insights [12]: McDonald’s was high in extraversion and agreeableness but low in openness and neuroticism, Harley-Davidson was high in openness and conscientiousness but low in neuroticism, and Tom’s Shoes was high in extraversion and agreeableness but low in neuroticism. In addition, the personalities of Indian celebrities [25] and mass murderers [27] were measured using IBM Watson Personality Insights as well. None of those, however, showed the same pattern in personality traits with the public health organizations reported in this study. This result can be helpful to understand the online behaviors of public health organizations from the perspective of personality, and further investigations are expected in future research concerning the difference in personality by various factors such as the type of organizations. In addition, online AI services can be actively employed to examine the personality of various non-human accounts such as governments, businesses, and nonprofits.

Next, it was found that each personality trait tended to be associated with certain kinds of photo features in particular directions. Openness and agreeableness were the personality traits that associated the most with the content-level features of the Instagram photos on public health organizations’ accounts, and their directions of association tended to be opposite to each other. In contrast, extraversion and neuroticism were the personality traits that were associated the most with the pixel-level features, and their directions of association tended to be opposite to each other. In the literature, personality traits have been found to be significantly associated with particular features of social media posts; for example, text features such as emotions [13], depth of self-disclosure [79], and linguistic markers [6] and profile photo features such as content [5] and facial and color features [78]. However, it is hard to find a pattern where one part of the personality traits was correlated mainly with content-level features and another part correlated mainly with pixel-level features and that, in each part, the directions of association of the traits tended to be opposite to each other. To the best of the authors’ knowledge, these results are reported for the first time in this study. Further research is expected to find out whether these results hold in the accounts of other kinds of organizations and to lay the theoretical foundation for understanding the relationship between the characteristics of visual data on organizational social media accounts and their personality.

In addition, the personality traits, except for neuroticism, of public health organizations’ Instagram accounts were predicted from the photo features with an acceptable level of accuracy. Given that the personality of individual users was shown to be predictable from their photo features [15,34], this result can be meaningful in that organizational accounts in social media can be investigated from the perspective of personality which has been employed for examining individual users’ accounts.

The results of correlational analysis also suggested that the organizational accounts were generally similar to individual users’ accounts in terms of the relationships between their personality and the features of their posts. For example, the correlations of happiness negatively with openness and positively with agreeableness, found in this study, are consistent with Schwartz et al. [13], Liu et al. [35], and Golbeck, Robles, and Turner [80] who analyzed the data from individual users’ accounts. The positive correlation between extraversion and the number of faces on photos can be also found in Celli, Bruni and Lepri [78], and Kim and Kim [20], and the negative correlation between openness and the number of faces on photos was also reported in Ferwerda, Schedl and Tkalcic [8], and Kim and Kim [20]. The positive correlations, reported in previous studies, of neuroticism with value mean [8] and hue peaks [20] were also obtained in this study. The positive correlation between conscientiousness and colorfulness can be also found in Liu et al. [35], while the positive correlation between neuroticism and brightness can be found in Ferwerda, Schedl, and Tkalcic [8]. Based on this correspondence, future research is expected to employ more various personality traits and other factors which have been used to explain online human behaviors for the investigation of organizational accounts.

The above findings can have implications from the perspective of public health. The online behaviors of public health organizations can be understood from the same perspective of personality as that of individual users of social media. Thus, the public can consider a social media campaign by public health organizations as communicating with an individual with a particular type of personality trait—more open and agreeable but less extravert and neurotic. Having this in mind, public health organizations can design social media messages which would better correspond with organizations’ personality. For example, the photo features that are associated with openness and agreeableness could be more stressed while the photo features that are associated with extraversion and neuroticism could be less stressed. This can make the social media messages from public health organizations more appealing, and future research is expected to test whether this would hold in the publics’ perception of the messages.

The major limitation of this study is that its research sample consisted of a limited number of public health organizations. Future study might include more Instagram accounts from diverse countries and cultures and compare them with each other. Also, a detailed analysis by the type of organizations is expected to be conducted in succeeding studies. How the public response might be different for the post features and platforms (e.g., Twitter and YouTube) would be a topic for future research as well.

## 6. Conclusions

The present study investigated the personality of Instagram accounts run by public health organizations and accordingly the differences of their uploaded photos in terms of content- and pixel-level characteristics. It identified the personality of public health organizations as being high in openness and agreeableness but low in extraversion and neuroticism. It was found that openness and agreeableness were the personality traits that associated the most with content-level features, while extraversion and neuroticism were the ones that associated the most with the pixel-level features. In addition, for each of the two traits that were associated the most with either content- or pixel- level features, their associations tended to be in opposing directions to one another. The personality traits, except neuroticism, were predicted from the photo features with an acceptable level of accuracy.

## Figures and Tables

**Table 1:** Instagram accounts of public health organizations in the research sample.

Category	Account Name
Federal Agencies	epagov, hhsgov, cdcgov, cdcglobal, actagainstaids, nioshusa, nationalservice, usdagov, hudgov, deptvetaffairs, nihgov, allofusresearch, nigms_nih, nichd_nih, niaid, niaaanews, nih_nccih, uscpsc, nationalcancerinstitute, nsvrc
State Health & Human Services	alabamapublichealth, azdhs, capublichealth, co_cdhs, dchealthdept, florida.health, healthy.ms, mnhealth, morh_rhi, nmdoh, njdeptofhealth, nysdoh, ohiodepartmentofhealth, oksoonercare, wadepthealth
Nonprofit Organizations	aarpphoto, adcouncil, advocatesforyouth, housingworks, alanon_wso, allergyasthmahq, alpha1foundation, alzassociation, herbalgramabc, americancancersociety, amdiabetesassn, amfar, amfmigraine, american_heart, cancerprevention, americankidneyfund, lungassociation, americanredcross, aspca, amputeecoalition, amyloidosisfoundation, angiomaalliance, annieecaseyfdn, triumphoveranxiety, apsfa, aadp_org, childhoodcancr, aafanational, b_c_a_n, blindchildrenscenter, bonemarrowfdn, boystownvillage, brailleinstitute, breakthecycle, breastcancerorg, cancercarepics, cancerhopenetwork, ccicanine, c_a_r_i_n_g, nationalceliac, cpaforg, solve_cfs, cwlaofficial, childrensbraintumorfoundation, ccfheartkids, codainternational, colorectalcanceralliance, thecompassionatefriends, cureepilepsy, cystinosisresearchfoundation, danafoundation, wefighteb, dbsalliance, davhq, disabledsportsusa, dogsforbetterlives, donatelifeamerica, dream_foundation, drugpolicyalliance, egpaf, fhi360, farmusa, fcsn_ma, feedingamerica, firstcandle, foodallergy, fracgram, facingourrisk, foundationforichthyosis, curepsp_, foundationforwomenscancer, futureswithoutviolence, gdb_official, guidedogsofamerica, guidingeyes, healthy_child, hearinglossassociation, helenkellerservices, hepbfoundation, safekidsworldwide, hopeafterloss, hdsanational, hydroassoc, ipfcc, theihi, ivatcenters, iihs_autosafety, dyslexiaida, icahelp, johntracyclinic, joslindiabetes, justiceinaging, kidneycancerassociation, kidspeacepa, imalivechatline, axysofficial, lamazechildbirth, leader_dog, ldaofamerica, lighthouseguild, livestrong, livingbeyondbc, lcaorg, lupusresearchalliance, lymphomacommunity, makeawishamerica, marchofdimes, mendedlittleheartsnational, menshealthnetwork, mentalhealthamerica, mercymedicalangels, miracleflights, themmrf, msassociation, multiple_sclerosis_foundation, mdaorg, namicommunicate, nad1880, natlbraintumorsociety, nbcf, powertodecide, missingkids, ncsbs, ncadv, national_homeless, natlcompcancernetwork, adoptioncouncil, nationalcouncil, weareunidosus, ndvhofficial, ndssorg, neda, nfaaware, togetherwalks, nfpadotorg, nfid_vaccines, nfxfoundation, nationalheadachefoundation, nihb1, nkcforg, the_nln, n.m.a.c, mpssociety, mssociety, noahalbinism, nocc_national, nrcdv, 1800runaway, sleepfoundation, nsda_sd, spinal_injuries, natlstrokeassoc, 800273talk, afspnational, touretteassociation, neweyes_, obesityaction, operationsmile, papaolalokahi, pva1946, parentprojectmd, petpartners, plannedparenthood, pkdfoundation, popconnectaction, preeclampsia.foundation, preventcancer, pathglobalhealth, projecthopeorg, projectinform, thepcri, pfforg, phassociation, rachelcarsondc, resolveorg, shapeamerica, scdaa, sjogrensfoundation, skincancerorg, seguidedogs, specialolympics, spinabifidaassn, spondylitis, thesturgeweberfoundation, stutteringfdn, tashorg, autismsociety, bafound, cmtausa, childrenstumor, clevelandclinic, kempefoundation, thelamfoundation, livingbank, oralcancerfoundation, thepartnership, progeriaresearch, seankimerling, theseeingeye, tlcbfrb, vbirthmarks, waismancenter, inc.officialtopsclub, tsalliance, unosnews, uoaa_, unitedway, urologycarefdn, vestibularveda, vhl_alliance, world_edu, zeroprostatecancer
Additional	americanpublichealth, pewenvironment, councilforchildrensrights, nationalkidneyfoundation, fightblindness, thecaregiverspace, bethematch, trustforpublicland, wildearthguardians, defendersofwildlife, leukaemia_foundation, projectpurple, sclerodermaus, postpartumprogress, womenshealthnetwork, unitedspinal, su2c, thejhf

**Table 2:** Correlations between public health organizations’ personality traits and content category of their Instagram photos.

Feature	Openness	Conscientiousness	Extraversion	Agreeableness	Neuroticism
abstract	0.300 *	−0.105	−0.123 *	−0.200 *	0.022
animal	0.070	−0.132 *	0.071	−0.006	0.095
building	0.142 *	−0.086	−0.008	−0.159 *	−0.056
dark	0.105	−0.034	−0.037	−0.049	0.057
drink	0.210 *	0.008	−0.021	−0.297 *	0.068
food	0.228 *	0.064	0.013	−0.320 *	0.028
indoor	−0.155 *	0.105	0.135 *	0.031	−0.037
others	0.208 *	−0.136 *	−0.013	−0.253 *	0.209 *
outdoor	0.074	−0.150 *	0.031	−0.204 *	−0.251 *
people	−0.269 *	0.000	0.070	0.246 *	−0.064
plant	0.323 *	−0.133 *	−0.046	−0.278 *	−0.103
object	0.103	−0.074	0.003	−0.158 *	−0.029
sky	0.120	−0.065	−0.033	−0.073	−0.046
text	−0.027	0.188 *	−0.063	0.125 *	0.074
transportation	−0.018	−0.129 *	−0.101	−0.248 *	−0.018
Gini	−0.334 *	0.115	0.010	0.357 *	0.011

* *p* < 0.05.

**Table 3:** Correlations between public health organizations’ personality traits and facial features of their Instagram photos.

Feature	Openness	Conscientiousness	Extraversion	Agreeableness	Neuroticism
number of faces	−0.304 *	0.156 *	0.184 *	0.353 *	−0.112
closeup	−0.062	−0.142 *	−0.058	0.274 *	0.057
face ratio	−0.116	−0.083	−0.016	0.315 *	0.029
age	−0.216 *	0.066	0.133 *	0.277 *	−0.079
gender	−0.262 *	0.150 *	0.222 *	0.349 *	−0.021
anger	−0.096	0.048	0.133 *	−0.006	−0.130 *
contempt	−0.150 *	−0.039	0.018	0.128 *	−0.066
disgust	−0.131 *	−0.023	0.079	0.019	−0.088
fear	−0.071	0.003	0.044	−0.006	0.026
happiness	−0.320 *	0.065	0.113	0.397 *	−0.049
neutral	−0.114	0.004	0.048	0.066	−0.113
sadness	−0.113	−0.060	0.019	−0.013	0.049
surprise	−0.189 *	0.049	0.059	0.125 *	−0.071

* *p* < 0.05.

**Table 4:** Correlations between public health organizations’ personality traits and pixel color features of their Instagram photos.

Feature	Openness	Conscientiousness	Extraversion	Agreeableness	Neuroticism
red mean	−0.019	0.079	−0.077	0.005	0.145 *
red var	−0.264 *	0.033	0.059	0.125 *	−0.009
green mean	0.013	0.025	−0.165 *	−0.062	0.188 *
green var	−0.241 *	0.078	0.134 *	0.112	−0.148 *
blue mean	−0.028	0.042	−0.184 *	0.012	0.196 *
blue var	−0.163 *	−0.002	0.042	−0.048	−0.195 *
saturation mean	0.071	0.075	0.064	−0.006	−0.116
saturation var	−0.095	0.096	−0.077	−0.087	−0.095
value mean	−0.007	0.090	−0.147 *	−0.004	0.174 *
value var	−0.188 *	−0.033	0.164 *	0.104	−0.161 *
red share	−0.046	0.099	0.151 *	0.222 *	−0.060
orange share	−0.007	−0.060	0.166 *	0.011	0.032
yellow share	0.079	0.100	0.233 *	−0.087	−0.064
green share	0.127 *	0.042	0.154 *	−0.011	−0.085
blue share	−0.033	−0.078	−0.202 *	−0.098	−0.017
violet share	-	-	-	-	-
share of warm colors	0.004	0.003	0.218 *	0.038	−0.007
share of cold colors	0.044	−0.050	−0.103	−0.100	−0.066
hue peaks	−0.024	−0.097	−0.096	−0.153 *	0.125 *

* *p* < 0.05.

**Table 5:** Correlations between public health organizations’ personality traits and visual features of their Instagram photos.

Feature	Openness	Conscientiousness	Extraversion	Agreeableness	Neuroticism
brightness	−0.001	0.045	−0.148 *	−0.035	0.184 *
colorfulness	−0.064	0.159 *	−0.035	−0.007	−0.055
naturalness	−0.011	0.119	0.192 *	−0.054	−0.175 *
contrast	−0.256 *	−0.011	0.107	0.073	−0.103
RGB_contrast	−0.258 *	0.033	0.090	0.061	−0.151 *
sharpness	0.057	−0.062	0.112	−0.093	−0.277 *
color_diversity	−0.069	0.028	0.076	−0.070	−0.099
color_harmony	−0.095	−0.080	−0.097	−0.075	−0.001
pleasure	0.011	0.115	−0.140 *	−0.006	0.156 *
arousal	0.054	0.010	0.117	−0.003	−0.168 *
dominance	0.025	−0.057	0.145 *	0.002	−0.183 *

* *p* < 0.05.

**Table 6:** Root mean square errors (RMSEs) in 10-fold cross validation of random forest regression on personality traits.

Feature	Openness	Conscientiousness	Extraversion	Agreeableness	Neuroticism
content category	0.117	0.131	0.146	0.161	0.195
facial features	0.120	0.132	0.149	0.160	0.201
pixel color features	0.118	0.132	0.145	0.167	0.194
visual features	0.121	0.129	0.143	0.170	0.198
all	0.115	0.131	0.141	0.158	0.199

## Data Availability

The data presented in this study are available on request from the corresponding author. The data are not publicly available due to privacy.

## References

[1] Uher J., Weiss A., King J.E., Murray L. (2011). Personality in nonhuman primates: What can we learn from human personality psychology. Personality and Temperament in Nonhuman Primates.

[2] Ryan T., Xenos S. (2011). Who uses Facebook? An investigation into the relationship between the Big Five, shyness, narcissism, loneliness, and Facebook usage. Comput. Hum. Behav..

[3] Seidman G. (2013). Self-presentation and belonging on Facebook: How personality influences social media use and motivations. Pers. Individ. Differ..

[4] Park G., Schwartz H.A., Eichstaedt J.C., Kern M.L., Kosinski M., Stillwell D.J., Ungar L.H., Seligman M.E.P. (2015). Automatic personality assessment through social media language. J. Pers. Soc. Psychol..

[5] Wu Y.-C.J., Chang W.-H., Yuan C.-H. (2015). Do Facebook profile pictures reflect user’s personality?. Comput. Hum. Behav..

[6] Kern M.L., Eichstaedt J.C., Schwartz H.A., Dziurzynski L., Ungar L.H., Stillwell D.J., Kosinski M., Ramones S.M., Seligman M.E.P. (2014). The online social self: An open vocabulary approach to personality. Assessment.

[7] Sorokowska A., Oleszkiewicz A., Frackowiak T., Pisanski K., Chmiel A., Sorokowski P. (2016). Selfies and personality: Who posts self-portrait photographs?. Pers. Individ. Differ..

[8] Ferwerda B., Schedl M., Tkalcic M., Tian Q., Sebe N., Qi G.-J., Huet B., Hong R., Liu X. (2016). Using Instagram picture features to predict users’ personality. Multimedia Modeling.

[9] Li L., Li A., Hao B., Guan Z., Zhu T. (2014). Predicting active users’ personality based on micro-blogging behaviors. PLoS ONE.

[10] Jordan P.W., Green W.S., Jordan P.W. (2002). The personality of products. Pleasure with Products: Beyond Usability.

[11] Park S.-E., Choi D., Kim J. (2005). Visualizing e-brand personality: Exploratory studies on visual attributes and e-brand personalities in Korea. Int. J. Hum. Comput. Stud..

[12] Yun J.T., Pamuksuz U., Duff B.R.L. (2019). Are we who we follow? Computationally analyzing human personality and brand following on Twitter. Int. J. Advert..

[13] Schwartz H.A., Eichstaedt J.C., Kern M.L., Dziurzynski L., Ramones S.M., Agrawal M., Shah A., Kosinski M., Stillwell D., Seligman M.E.P. (2013). Personality, gender, and age in the language of social media: The open-vocabulary approach. PLoS ONE.

[14] Qiu L., Lin H., Ramsay J., Yang F. (2012). You are what you tweet: Personality expression and perception on Twitter. J. Res. Pers..

[15] Al-Kandari A.A., Gaither T.K., Alfahad M.M., Dashti A.A., Alsaber A.R. (2019). An Arab perspective on social media: How banks in Kuwait use instagram for public relations. Public Relat. Rev..

[16] Cassinger C., Thelander Å. (2020). Voicing the organization on Instagram: Towards a performative understanding of employee voice. Public Relat. Inq..

[17] Kharmalki G.W., Raizada S. (2020). Social media marketing in sports: The rise of fan engagement through Instagram. Ann. Trop. Med. Health.

[18] Filimonov K., Russmann U., Svensson J. (2016). Picturing the party: Instagram and party campaigning in the 2014 Swedish elections. Soc. Media Soc..

[19] Eftekhar A., Fullwood C., Morris N. (2014). Capturing personality from Facebook photos and photo-related activities: How much exposure do you need?. Comput. Hum. Behav..

[20] Kim Y., Kim J.H. (2018). Using computer vision techniques on Instagram to link users’ personalities and genders to the features of their photos: An exploratory study. Inf. Process. Manag..

[21] Kim J.H., Kim Y. (2019). Instagram user characteristics and the color of their photos: Colorfulness, color diversity, and color harmony. Inf. Process. Manag..

[22] Li A., Yan Z., Zhu T. (2013). Self-report versus web-Log: Which one is better to predict personality of website users?. Int. J. Cyber Behav. Psychol. Learn..

[23] Youyou W., Kosinski M., Stillwell D. (2015). Computer-based personality judgments are more accurate than those made by humans. Proc. Natl. Acad. Sci. USA.

[24] Balakrishnan V., Khan S., Fernandez T., Arabnia H.R. (2019). Cyberbullying detection on twitter using Big Five and Dark Triad features. Pers. Individ. Differ..

[25] Dutta K., Singh V.K., Chakraborty P., Sidhardhan S.K., Krishna B.S., Dash C. (2017). Analyzing Big-Five personality traits of Indian celebrities using online social media. Psychol. Stud..

[26] Kern M.L., McCarthy P.X., Chakrabarty D., Rizoiu M.-A. (2019). Social media-predicted personality traits and values can help match people to their ideal jobs. Proc. Natl. Acad. Sci. USA.

[27] Kop M., Read P., Walker B.R. (2019). Pseudocommando mass murderers: A big five personality profile using psycholinguistics. Curr. Psychol..

[28] Whittingham N., Boecker A., Grygorczyk A. (2020). Personality traits, basic individual values and GMO risk perception of twitter users. J. Risk Res..

[29] Digman J.M. (1990). Personality structure: Emergence of the five-factor model. Annu. Rev. Psychol..

[30] McCrae R.R., John O.P. (1992). An introduction to the five-factor model and Its applications. J. Pers..

[31] Pentina I., Zhang L. (2017). Effects of social support and personality on emotional disclosure on Facebook and in real life. Behav. Inform. Technol..

[32] Miller R.E. (2020). College students and inappropriate social media posting: Is it a question of personality or the influence of friends. Pers. Individ. Differ..

[33] Wang S., Chen X. (2020). Recognizing CEO personality and its impact on business performance: Mining linguistic cues from social media. Inf. Manag..

[34] Agarwal A., Toshniwal D. (2020). Identifying leadership characteristics from social media data during natural hazards using personality traits. Sci. Rep..

[35] Liu L., Preotiuc-Pietro D., Samani Z.R., Moghaddam M.E., Ungar L.H. Analyzing personality through social media profile picture choice. Proceedings of the 10th International AAAI Conference on Web and Social Media (ICWSM-16).

[36] Samani Z.R., Guntuku S.C., Moghaddam M.E., Preoţiuc-Pietro D., Ungar L.H. (2018). Cross-platform and cross-interaction study of user personality based on images on Twitter and Flickr. PLoS ONE.

[37] Uher J. (2008). Comparative personality research: Methodological approaches. Eur. J. Pers..

[38] Govers P.C.M., Schoormans J.P.L. (2005). Product personality and its influence on consumer preference. J. Consum. Mark..

[39] Liao I.-C., Deng Y.-S., You H.-C., Marcus A. (2016). The emotion and personality user perception in multi-screen interaction. Design, User Experience, and Usability: Novel User Experiences.

[40] D’astous A., Lévesque M. (2003). A scale for measuring store personality. Psychol. Mark..

[41] Caprara G.V., Barbaranelli C., Guido G. (2001). Brand personality: How to make the metaphor fit. J. Econ. Psychol..

[42] Eisend M., Stokburger-Sauer N.E. (2013). Measurement characteristics of Aaker’s brand personality dimensions: Lessons to be learned from human personality research. Psychol. Mark..

[43] Wee T.T.T. (2004). Extending human personality to brands: The stability factor. J. Brand Manag..

[44] Geuens M., Weijters B., De Wulf K. (2009). A new measure of brand personality. Int. J. Res. Mark..

[45] Mulyanegara R.C., Tsarenko Y., Anderson A. (2009). The Big Five and brand personality: Investigating the impact of consumer personality on preferences towards particular brand personality. J. Brand Manag..

[46] Chen Q., Rodgers S. (2006). Development of an instrument to measure web site personality. J. Interact. Advert..

[47] Poddar A., Donthu N., Wei Y. (2009). Web site customer orientations, web site quality, and purchase intentions: The role of web site personality. J. Bus. Res..

[48] Shobeiri S., Laroche M., Mazaheri E. (2013). Shaping e-retailer’s website personality: The importance of experiential marketing. J. Retail. Consum. Serv..

[49] Shobeiri S., Mazaheri E., Laroche M. (2015). How would the e-retailer’s website personality impact customers’ attitudes toward the site. J. Mark. Theory Pract..

[50] Akrimi Y., Khemakhem P.R. (2014). An analysis of perceived usability, perceived interactivity and website personality and their effects on consumer satisfaction. Int. J. Manag. Excell..

[51] Akrimi Y. (2016). Usability, interactivity, website personality and consumers’ responses: A case of internet service provider. Int. J. Electron. Mark. Retail..

[52] Rezaei S., Ali F., Amin M., Jayashree S. (2016). Online impulse buying of tourism products: The role of web site personality, utilitarian and hedonic web browsing. J. Hosp. Tour. Technol..

[53] Jain K., Yadav D., Kapur P.K., Klochkov Y., Verma A.K., Singh G. (2019). The role of website personality and website user engagement on individual’s purchase intention. System Performance and Management Analytics: Asset Analytics.

[54] Moon Y., Nass C. (1996). How “real” are computer personalities? Psychological responses to personality types in human-computer interaction. Commun. Res..

[55] Hinds J., Joinson A. (2019). Human and computer personality prediction from digital footprints. Curr. Dir. Psychol. Sci..

[56] Moreno-Armendariz M.A., Duchanoy Martinez C.A., Calvo H., Moreno-Sotelo M. (2020). Estimation of personality traits from portrait pictures using the five-factor model. IEEE Access.

[57] Park H., Rodgers S., Stemmle J. (2011). Health organizations’ use of Facebook for health advertising and promotion. J. Interact. Advert..

[58] Guidry J.P.D., Jin Y., Orr C.A., Messner M., Meganck S. (2017). Ebola on Instagram and Twitter: How health organizations address the health crisis in their social media engagement. Public Relat. Rev..

[59] Steffens M.S., Dunn A.G., Wiley K.E., Leask J. (2019). How organisations promoting vaccination respond to misinformation on social media: A qualitative investigation. BMC Public Health.

[60] Chen Q., Min C., Zhang W., Wang G., Ma X., Evans R. (2020). Unpacking the black box: How to promote citizen engagement through government social media during the COVID-19 crisis. Comput. Hum. Behav..

[61] Liao Q., Yuan J., Dong M., Yang L., Fielding R., Lam W.W.T. (2020). Public engagement and government responsiveness in the communications about COVID-19 during the early epidemic stage in China: Infodemiology study on social media data. J. Med. Internet Res..

[62] Kim Y., Kim J.H. (2020). Using photos for public health communication: A computational analysis of the Centers for Disease Control and Prevention Instagram photos and public responses. J. Health Inform..

[63] Ferwerda B., Tkalcic M. You are what you post: What the content of Instagram pictures tells about users’ personality. Proceedings of the Second Workshop on Theory-Informed User Modeling for Tailoring and Personalizing Interfaces (HUMANIZE).

[64] Del Sole A. (2018). Microsoft Computer Vision APIs Distilled: Getting Started with Cognitive Services.

[65] Ke Y., Tang X., Jing F. The design of high-level features for photo quality assessment. Proceedings of the 2006 IEEE Computer Society Conference on Computer Vision and Pattern Recognition.

[66] Mao X., Chen B., Muta I. (2003). Affective property of image and fractal dimension. Chaos Solitons Fractals.

[67] San Pedro J., Siersdorfer S. Ranking and classifying attractiveness of photos in folksonomies. Proceedings of the 18th International Conference on World Wide Web (WWW’09).

[68] Hasler D., Süsstrunk S., Rogowitz B.E., Pappas T.N. (2003). Measuring colorfulness in natural images. Human Vision and Electronic Imaging VIII..

[69] Huang K.-Q., Wang Q., Wu Z.-Y. (2006). Natural color image enhancement and evaluation algorithm based on human visual system. Comput. Vis. Image Underst..

[70] Savakis A.E., Etz S.P., Loui A.C., Rogowitz B.E., Pappas T.N. (2000). Evaluation of image appeal in consumer photography. Human Vision and Electronic Imaging V..

[71] Kim D., Son S.-W., Jeong H. (2014). Large-scale quantitative analysis of painting arts. Sci. Rep..

[72] Feng J., Lin W.-C., Chen C.-T. Fractional box-counting approach to fractal dimension estimation. Proceedings of the 13th International Conference on Pattern Recognition.

[73] Moon P., Spencer D.E. (1944). Geometric formulation of classical color harmony. J. Opt. Soc. Am..

[74] Datta R., Joshi D., Li J., Wang J.Z., Leonardis A., Bischof H., Pinz A. (2006). Studying aesthetics in photographic images using a computational approach. Computer Vision: ECCV 2006.

[75] Valdez P., Mehrabian A. (1994). Effects of color on emotions. J. Exp. Psychol. Gen..

[76] Quercia D., Kosinski M., Stillwell D., Crowcroft J. Our Twitter profiles, our selves: Predicting personality with Twitter. Proceedings of the 2011 IEEE Third International Conference on Privacy, Security, Risk and Trust, and 2011 IEEE Third International Conference on Social Computing.

[77] Skowron M., Tkalčič M., Ferwerda B., Schedl M. Fusing social media cues: Personality prediction from Twitter and Instagram. Proceedings of the 25th International Conference Companion on World Wide Web (WWW ‘16 Companion).

[78] Celli F., Bruni E., Lepri B. Automatic personality and interaction style recognition from Facebook profile pictures. Proceedings of the ACM International Conference on Multimedia—MM’14.

[79] Winter S., Neubaum G., Eimler S.C., Gordon V., Theil J., Herrmann J., Meinert J., Krämer N.C. (2014). Another brick in the Facebook wall: How personality traits relate to the content of status updates. Comput. Hum. Behav..

[80] Golbeck J., Robles C., Turner K. Predicting personality with social media. Proceedings of the ACM CHI Conference on Human Factors in Computing Systems (CHI 2011).

